# The *Agrobacterium* VirD5 protein hyperactivates the mitotic Aurora kinase in host cells

**DOI:** 10.1111/nph.15700

**Published:** 2019-03-02

**Authors:** Xiaorong Zhang, Paul J. J. Hooykaas

**Affiliations:** ^1^ Department of Molecular and Developmental Genetics Institute of Biology Leiden University Sylviusweg 72 Leiden 2333BE the Netherlands

**Keywords:** *Agrobacterium tumefaciens*, Aurora kinase, kinetochore, mitosis, virulence

## Abstract

- Aided by translocated virulence proteins, *Agrobacterium tumefaciens* transforms plant cells with oncogenic T‐DNA. In the host cells the virulence protein VirD5 moves to the nucleus, where it becomes localized at the kinetochores, and disturbs faithful chromosome segregation, but the molecular mechanism underlying this remains unknown.
- To gain more insight, we screened amongst the kinetochore proteins for VirD5 interactors using bimolecular fluorescence complementation assays, and tested chromosome segregation in yeast cells.
- We found that VirD5 interacts with the conserved mitotic Aurora kinase Ipl1 in yeast and likewise with plant Aurora kinases. *In vitro* VirD5 was found to stimulate the activity of Ipl1. Phosphorylation of substrates by Ipl1 *in vivo* is known to result in the detachment between kinetochore and spindle microtubule. This is necessary for error correction, but increased Ipl1/Aurora kinase activity is known to cause spindle instability, explaining enhanced chromosome mis‐segregation seen in the presence of VirD5. That activation of the Ipl1/Aurora kinase at least partially underlies the toxicity of VirD5 became apparent by artificial boosting the activity of the specific counteracting phosphatase Glc7 *in vivo*, which relieved the toxicity.
- These findings reveal a novel mechanism by which a pathogenic bacterium manipulates host cells.

## Introduction

The soil bacterium *Agrobacterium tumefaciens* transforms plant cells into tumor cells by the delivery of an oncogenic piece of DNA from its Ti (tumor‐inducing) plasmid (Nester *et al*., 1984; Tzfira *et al*., 2000; Gelvin, 2003); nowadays, it is an important gene vector not only for plants but also for fungi (Michielse *et al*., 2005). Transformation is facilitated by the translocation of a set of virulence (effector) proteins into the host cells by the bacterial Type4 Secretion System (T4SS). These virulence proteins are involved in protection and nuclear delivery of the transferred DNA molecules (Tzfira *et al*., 2000; Gelvin, 2003), but also in mitigating host defense responses (Garcia‐Cano *et al*., 2015; Niu *et al*., 2015). One of these effector proteins, VirD5, is a large protein of 833 amino acids (aa) with multiple nuclear localization signals (NLSs) and several other motifs (Schrammeijer *et al*., 2000). VirD5 binds to and stabilizes another translocated effector protein called VirF (Magori & Citovsky, 2011). VirD5 has also been shown to compete with the host protein VBF for binding to host protein VIP1 (Wang *et al*., 2014), and recently it was shown by the same group that it also competes with host protein CBP20 for binding to host protein VIP2 (Wang *et al*., 2018). Studies of VirD5 are difficult because constitutive expression of VirD5 in host cells is toxic and may lead to cell death (Zhang *et al*., 2017). In the nucleus VirD5 shows a punctate localization, which is dependent on interaction of the N‐terminal part of VirD5 (VirD5NT) with the host Spt4 protein (Zhang *et al*., 2017). The Spt4 protein partially localizes to the centromeres/kinetochores (Crotti & Basrai, 2004), and it was found that VirD5 colocalizes at the centromeres/kinetochores with Spt4. In the absence of Spt4, VirD5 no longer localizes at the centromeres/kinetochores and has strongly reduced toxicity. The presence of VirD5 at the kinetochores affects mitosis and causes chromosome mis‐segregation in both budding yeast and plants (Zhang *et al*., 2017).

The kinetochores are responsible for the faithful segregation of the duplicated chromosomes over the daughter cells during mitosis (Cheeseman & Desai, 2008; Biggins, 2013; Yamagishi *et al*., 2014). Structurally, kinetochores consist of an inner kinetochore, which is bound directly to the centromere, and an outer kinetochore, which mediates spindle microtubule attachment. Chromosomes are segregated over daughter cells as the spindle microtubules depolymerize and shorten at the onset of anaphase (Kline‐Smith & Walczak, 2004). In budding yeast, a 50‐nm ring‐like structure consisting of the 10 proteins of the Dam1 complex embraces the microtubules, and directly connects to outer kinetochore proteins of the Ndc80 complex (Miranda *et al*., 2005; Zelter *et al*., 2015). As the Dam1 ring complex remains attached to the tips of spindle microtubules during the depolymerization of microtubules, it facilitates chromosome movement in anaphase to the cell poles (Westermann *et al*., 2006; Umbreit *et al*., 2014). For faithful chromosome segregation, sister kinetochores must attach to spindle microtubules emanating from the two opposite poles (Tanaka, 2005; Foley & Kapoor, 2013). Incorrect attachments provoke the spindle assembly checkpoint (SAC), which arrests cells in metaphase until all duplicated chromosomes are bipolarly attached (Sacristan & Kops, 2015). Correction of improper kinetochore–microtubule attachments is achieved by the Aurora B kinase, called Ipl1 in budding yeast (Biggins *et al*., 1999; Saurin *et al*., 2011). The Ipl1/Aurora B kinase destabilizes erroneous attachments via phosphorylation of key proteins including Ndc80 and Dam1 that are involved directly in the kinetochore–microtubule attachment (Tien *et al*., 2010; DeLuca *et al*., 2011; Umbreit *et al*., 2014). Therefore, a reduction in the activity of Aurora B/Ipl1 leads to chromosome mis‐segregation and aneuploidy. Overexpression of Aurora B/Ipl1, however, also leads to defective chromosome segregation, as the repeated disruption of kinetochore–microtubule attachments not only activates the SAC, but in the end generates lagging chromosomes and aneuploid cells (Muñoz‐Barrera & Monje‐Casas, 2014).

Here we investigated the molecular basis underlying the toxicity and mode of action of the *Agrobacterium* virulence protein VirD5, which becomes bound to the kinetochores via an interaction with the host Spt4 protein (Zhang *et al*., 2017). We screened amongst the kinetochore proteins for VirD5 interactors and found that at the kinetochores VirD5 interacts with the conserved Ipl1/Aurora kinase in both yeast and plants. By enhancing the activities of this conserved kinase, VirD5 delays mitotic progression and causes chromosome mis‐segregation and aneuploidy.

## Materials and Methods

### Strains, plasmids and primers

The strains, plasmids and primers used in this study are described in Supporting Information Tables S1–S3. Throughout we used the *virD5* coding sequence from the octopine strain B6; for comparison we also performed experiments with *virD5* from nopaline strain C58 and from the limited‐host‐range strain AB2/73.

### Root transformation

Root transformation was performed as described by Vergunst *et al*. (2005). Root segments were infected with either tumorigenic *Agrobacterium* strains LBA1010 or LBA3550 (the *virD5* deletion mutant) or with the corresponding helper strains lacking the oncogenic T‐DNA, but carrying pCAMBIA 3301 instead. After cocultivationon on callus induction medium containing 100 μM acetosyringone for 48 h, root segments were washed, dried and incubated on shoot induction medium supplemented with 30 μg ml^−1^ phosphinothricin, 500 μg ml^−1^ carbenicillin and 100 μg ml^−1^ vancomycin. After 3–4 wk, plates were photographed and transformation efficiencies were scored as infected root segments that produced any form of green callus. Statistical analyses were performed using prism v.5 (GraphPad Software Inc., La Jolla, CA, USA). Our results confirmed the results described by Wang *et al*. (2018) showing a reduced transformation by both of the *virD5* mutant strains (Fig. S1).

### BIFC assay

The pUG34VCn‐VirD5 plasmid (or pUG34VCn‐VirD5NT or pUG34VCn‐VirD5CT) was transformed either with pUG35VNc‐Dam1 or pUG35VNc‐Ipl1 into wild‐type yeast cells. Transformants were grown at 30°C on solid minimal MY (minimal) medium containing 30 mg l^−1^ methionine to inhibit the expression of VirD5 or its truncations. After 3 d, colonies were transferred to MY liquid medium containing 30 mg l^−1^ methionine. Overnight cultures washed twice with sterilized water were transferred into new flasks containing MY medium lacking methionine to induce the expression of VirD5 or its truncations. After induction for 1 h, cells were harvested for bimolecular fluorescent complementation (BIFC) signal (excitation, 514 nm; emission, 522–532 nm) visualization using a ×63 oil objective on the Zeiss Imager confocal microscope. Images were processed with imagej (National Institutes of Health, Bethesda, MD, USA) and Photoshop(Adobe). Yeast strains and plasmids used in this study are listed in Tables S1 and S2, respectively.

### *In vitro* kinase assay

His‐tagged Ipl1 and His‐tagged VirD5 were expressed and purified with Ni‐NTA Agarose (Qiagen: cat. 30310). GST‐tagged Dam1 protein was expressed in *Escherichia coli* strain Rosette2PLySs. Cell cultures of 50 ml were centrifuged and resuspended in 1 ml lysis buffer (50 mM NaH_2_PO_4_, 150 mM NaCl, pH 7.2, 1 mM DTT, 1 mM EDTA, 1% Triton X‐100). After sonication and centrifugation, the supernatants were stored at −80°C. The kinase assay was performed (Keating *et al*., 2009) by incubating GST‐Dam1 (10 μl supernatant from 1 ml lysate‐bound glutathione beads), 1 μl purified His‐Ipl1 and 8 μl purified His‐VirD5 in buffer containing 50 mM Tris‐HCl (pH 7.5), 0.1% (v/v) β‐mercaptoethanol, 0.1 mM EGTA, 10 mM MgCl_2_, 100 μM ATP and 1 μCi of [γ‐^32^P] ATP for 1 h at 30°C. The reaction was stopped by adding 5 μl 4× sample buffer and boiling for 10 min and the mixture was loaded on a 10% SDS‐PAGE gel and processed for autoradiography or Coomassie Brilliant Blue staining to confirm the equivalent loading of GST‐Dam1 protein.

### Pull‐down assay

GST, GST‐Dam1, GST‐Ipl1, GST‐Ipl1NT (1–118 aa) and GST‐Ipl1CT (101–367 aa) were expressed in *E. coli* strain Rosette2PLySs. Equal amounts of the GST‐tagged proteins were immobilized on Glutathione HiCap Matrix (Qiagen: cat. 30900) for 2 h at room temperature, followed by three washing steps with washing buffer (50 mM NaH_2_PO_4_, 150 mM NaCl, pH 7.2, 1 mM DTT, 1 mM EDTA). The beads were incubated with purified His‐tagged VirD5 protein in binding buffer (50 mM NaH_2_PO_4_, 150 mM NaCl, pH 7.2, 0.1% Triton X‐100) for 2 h at room temperature. After three washes with buffer (50 mM Tris, pH 8.0, 200 mM NaCl, 1 mM DTT, 1 mM EDTA, 10 mM MgCl_2_, 1% Nonidet P‐40), samples were mixed with 20 μl 4× sample buffer and boiled for 10 min, followed by centrifugation for 2 min at 376 ***g***. Supernatants were loaded on a 10% SDS‐PAGE gel for electrophoresis. The presence of the His‐tagged VirD5 protein was detected with Anti‐His HRP antibodies (Santa Cruz Biotechnology, Santa Cruz, CA, USA, sc‐8036 HRP) by Western blot analysis.

### Chromosome loss assay

Strain RLY4029 (a kind gift from Dr Rong Li, Baltimore) contains a minichromosome consisting of a fragment of yeast chromosome III, with the *SUP11* and *URA3* marker genes (Chen *et al*., 2012). The genetic background of this haploid strain carries an *ade2‐101* mutation and therefore forms red colonies in the absence of the minichromosome. The red pigment accumulation, however, is suppressed by the expression of *SUP11* present on the minichromosome, resulting in white colonies. The frequency of loss of this minichromosome can be calculated by counting the numbers of red colonies among the total numbers of colonies (Fig. S2). Wild‐type cells were transformed with either high‐copy plasmid (pRS425‐HYG) or its derivative (pRS425‐HYG‐VirD5NT‐3xNLS) encoding VirD5NT‐3xNLS under control of the *GAL1* promoter. These strains can be propagated without loss of the minichromosome on MY medium with 2% glucose, but without leucine and uracil. For analysis of minichromosome loss, cells were first cultured overnight in MY glucose medium lacking uracil at 30°C and then diluted and recultured in MY glucose liquid media without uracil for an additional 6 h. Cells were then diluted 50‐fold and switched to rich medium YP (10 g l^−1^ yeast extract, 20 g l^−1^ Bacto peptone) containing hygromycin (200 μg ml^−1^), 2% raffinose and 2% galactose for 24 h at 30°C to induce VirD5NT‐3xNLS. Overnight cultured cells were diluted to an appropriate density and plated onto rich medium YP containing hygromycin and 2% glucose for 3 d at 30°C. Plates were kept at 4°C for accumulation of red pigment. Total white and red colony numbers were counted.

### Chromosome segregation assay

Yeast strain Y716 (a gift from Dr Dean Dawson, Oklahoma City) contains a gene for expressing GFP‐LacI and a 256 repeat *lacO* array integrated in chromosome I. Enrichment of GFP‐LacI at the *lacO* repeats allows visualization of chromosome I as a fluorescent dot. Wild‐type cells transformed with either high‐copy plasmid (pRS425‐HYG) or its derivative (pRS425‐HYG‐VirD5NT‐3xNLS) encoding VirD5NT‐3xNLS under control of the *GAL1* promoter were cultured in YP (10 g l^−1^ yeast extract, 20 g l^−1^ peptone) rich medium containing 2% glucose and hygromycin (200 μg ml^−1^). Overnight cultured cells were diluted to an OD_620_ of 0.1 and recultured in YP rich medium containing hygromycin, 2% raffinose and 2% galactose for an additional 6 h. A GFP fluorescent dot (excitation, 488 nm; emission, 520 nm) was visualized via a ×63 oil objective on a Zeiss Imager confocal microscope. One hundred anaphase cells were analyzed in each experiment. Images were processed with imagej and photoshop.

### Protoplast transformation

Protoplast transformation was performed following the protocol of Schirawski *et al*. (2000) with slight modifications as follows. In total, 10 μg of DNA for each plasmid was used in a single transformation. After adding the PEG solution (40% PEG 4000, 0.2 M mannitol, 0.1 M CaCl_2_) the transformed cells were transferred 10 min later to the plates containing protoplast medium. After 30 min, the plates were sealed and incubated overnight at 25°C in the dark for 18 h. BIFC signal (excitation, 514 nm; emission, 522–532 nm) was visualized using a ×63 oil objective on a Zeiss Imager confocal microscope. Images were processed with imagej and photoshop.

### Benomyl sensitivity assay

Single‐copy plasmid pRS315 or pRS315‐VirD5NT or high‐copy plasmid pRS425 or pRS425‐VirD5NT was transformed into wild‐type BY4743 yeast cells. Transformants were grown at 30°C on solid minimal MY medium containing 2% glucose, uracil and histidine. After 3 d, colonies were streaked onto new solid minimal MY medium containing 2% glucose, uracil and histidine and were incubated overnight at 30°C. Positive colonies were resuspended in sterile water and were serially diluted five‐fold and spotted onto solid minimal MY medium containing either 2% glucose or 2% galactose with or without 15 μg ml^−1^ benomyl. After 3 d of incubation at 30°C, photos of the colonies were made and processed with photoshop.

### Sequence alignment

Protein sequences of yeast Ipl1 (NP_015115), *Arabidopsis thaliana* Aurora 1 (NP_195009), Aurora 2 (NP_180159), Aurora 3 (NP_182073), and human AurkA (NP_003591), AurkB (NP_001271455) and AurkC (NP_001015878) were downloaded from NCBI (https://www.ncbi.nlm.nih.gov/) and were imported into clc software (CLC workbench, Qiagen). After alignment, the image was exported and further processed with photoshop.

### Plant material

Binary vector pGPINTAM‐VirD5NT (1–505 aa) containing *virD5NT* under control of a tamoxifen inducible promoter was transferred into *A. tumefaciens* strain AGL1. *Arabidopsis thaliana* ecotype Columbia‐0 (Col‐0) was used for floral dip transformation (Clough & Bent, 1998). Mature seeds were harvested and sowed on MS medium (2.3 g l^−1^ MS medium including vitamins (Duchefa), 0.5 g l^−1^ MES, 7 g l^−1^ agar, pH 5.8) containing 50 mg l^−1^ kanamycin. Kanamycin‐resistant T1 transgenic seedlings were transferred to soil. T2 seeds from three independent T1 transgenic plants were germinated on MS medium containing kanamycin and either DMSO or 10 μM tamoxifen to induce the expression of VirD5NT.

## Results

### VirD5 interacts with the kinetochore‐associated Aurora kinase Ipl1 in yeast

**Figure 1:**
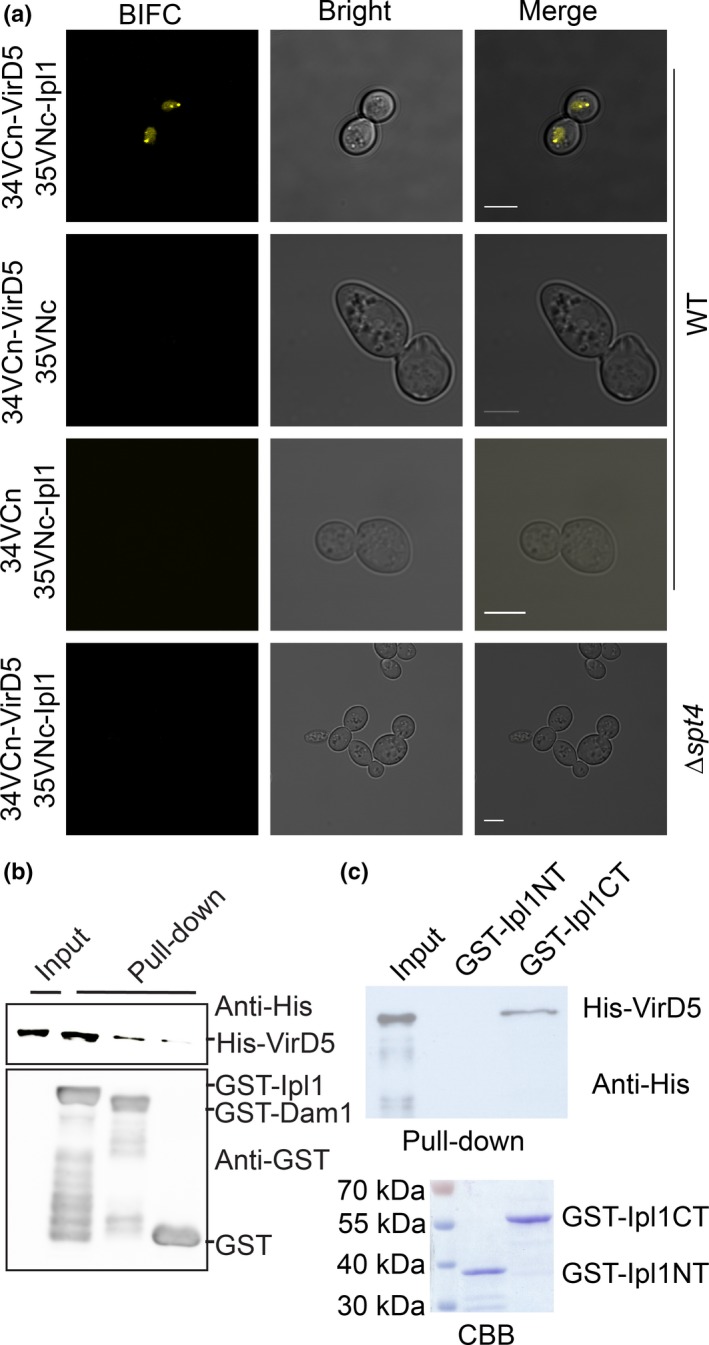
VirD5 interacts with the yeast Aurora kinase Ipl1. (a). The wild‐type (WT) yeast strain BY4743 or the *spt4* mutant was transformed with BIFC vectors. 34VCn, the C‐terminus of Venus (VC173) fused with the N‐terminus of the proteins to be tested. 35VNc, the N‐terminus of Venus (VN173) fused with the C‐terminus of the proteins to be tested. Bars, 5 μm. (b) His‐tagged VirD5 purified from *Escherichia coli* was incubated with either empty GST or GST‐tagged Dam1 or Ipl1 *in vitro*; after washing, the presence of His‐VirD5 bound to the glutathione HiCap matrix was detected by anti‐His antibody (His‐probe antibody, Santa Cruz). Top from left to right: input His‐VirD5; pulldown His‐VirD5 by GST‐Ipl1; pulldown His‐VirD5 by GST‐Dam1; pulldown His‐VirD5 by GST. Bottom protein samples obtained from *E. coli*, from left to right: no protein input; GST‐Ipl1; GST‐Dam1; GST. Extra bands represent impurities still present after purification. (c) His‐tagged VirD5 was incubated with either GST‐Ipl1NT or GST‐Ipl1CT *in vitro*; after washing, the presence of His‐VirD5 bound to the glutathione HiCap matrix was detected by anti‐His antibody. Ipl1NT, the N‐terminal part of Ipl1 (1–118 aa). Ipl1CT, the C‐terminal catalytic part of Ipl1 (101–367 aa).

We showed recently that the *Agrobacterium* virulence protein VirD5 is located at the kinetochores in host cells and causes chromosome mis‐segregation (Zhang *et al*., 2017). Binding to the kinetochores is dependent on an interaction of the N‐terminal part of VirD5 (Zhang *et al*., 2017) with the host kinetochore‐localized Spt4 protein (Crotti & Basrai, 2004). Here we studied whether VirD5 interacts not only with Spt4, but also with other proteins at the kinetochore. To this end, we performed BIFC experiments (Kerppola, 2008) with a set of candidate proteins (Table S4) from the inner and outer kinetochore of yeast, many of which are conserved in animals and plants. These were fused with the N‐terminal part of the improved yellow fluorescent protein (YFP) called Venus (VN173), and introduced into yeast cells together with VirD5 fused with the C‐terminal part of Venus (VC173). The outer kinetochore/microtubule‐associated protein Dam1 and the essential mitosis regulatory Ipl1/Aurora kinase displayed a strong BIFC YFP signal with VirD5 (Fig. 1a, S3), while no YFP signal was observed in the controls (Fig. 1a, S3). To confirm this result, which we obtained with the VirD5 protein from the octopine strain B6, we also tested the same interactions with VirD5 from two other *Agrobacterium* strains, namely from the nopaline strain C58 and from the limited‐host‐range strain AB2/73. These VirD5 proteins interacted positively with both Ipl1 and Dam1 (Fig. S4). No interactions of VirD5 with any of the other tested kinetochore proteins were observed (Table S4).

Previously, we found that after deletion of the *spt4* gene, VirD5 was no longer present as punctate foci at the kinetochores in the nucleus, but distributed over the entire nucleus (Zhang *et al*., 2017). Therefore, we tested whether the interactions of VirD5 with Dam1 and Ipl1 relied on the presence of Spt4 in the cell. To this end we repeated the BIFC experiment in the *spt4* deletion mutant. As shown in Fig. 1(a) and Fig. S3, no YFP signal was observed in *spt4* mutant cells expressing VirD5 together with either Dam1 or Ipl1, demonstrating that the interaction with Dam1 and Ipl1 is dependent on Spt4. Nevertheless, in *in vitro* pull‐down assays in *E. coli* of His‐tagged VirD5 with Ipl1 and Dam1, bound as GST fusion proteins to a Glutathione HiCap Matrix, VirD5 was recovered particularly in the case of Ipl1 (Fig. 1b). No VirD5 was recovered from beads bound by empty GST, which suggests that VirD5 may interact directly with Ipl1. The direct binding of VirD5 to the beads with Ipl1 seen *in vitro* may be due to high protein concentrations, obviating a need for Spt4. This interaction, however, apparently is too weak to attract VirD5 *in vivo* to the kinetochore in the absence of Spt4.

### VirD5 binds also to plant Aurora kinases

**Figure 2:**
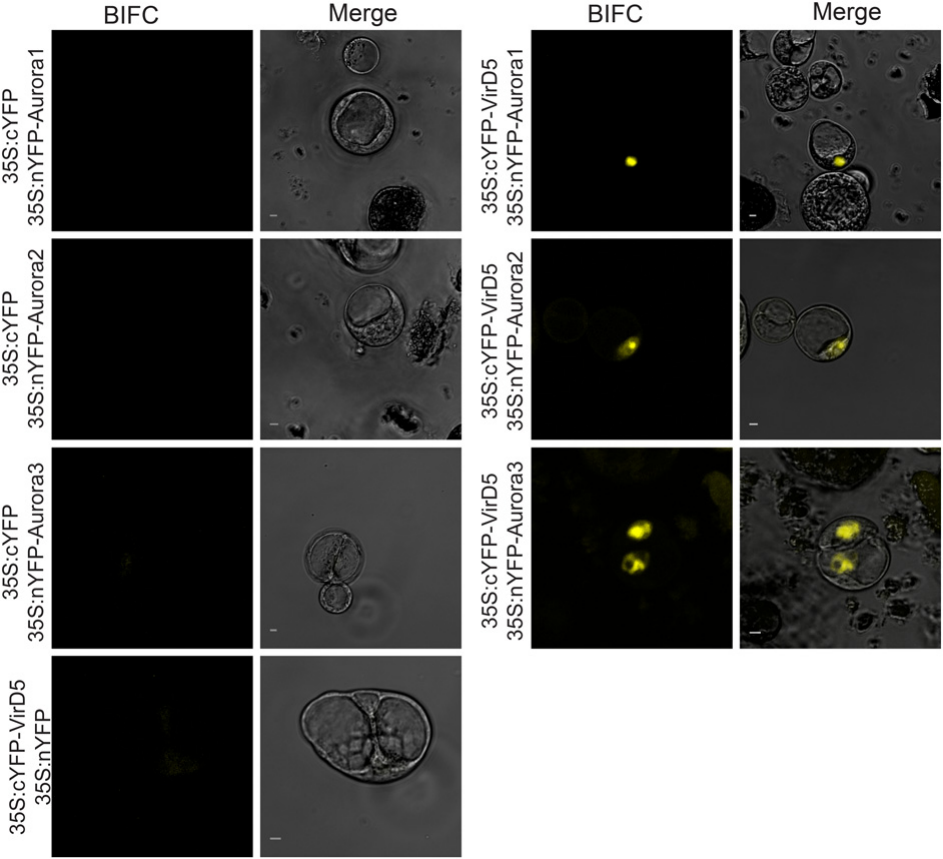
VirD5 interacts with three *Arabidopsis thaliana* Aurora kinases. *A*. *thaliana* protoplasts were transformed with plant BIFC vectors. 35S, Cauliflower mosaic virus promoter. nYFP, N‐terminus of YFP (1–154 aa). cYFP, C‐terminus of YFP (155–238 aa). Bars, 5 μm.

The Ipl1 protein belongs to the conserved Aurora serine/threonine protein kinase family (Fig. S5), which plays an essential role in the control of appropriate kinetochore–microtubule attachments during mitosis in eukaryotes (Andrews *et al*., 2003; Buvelot *et al*., 2003; Demidov *et al*., 2014). An interaction of VirD5 with Aurora kinases in yeast and plants may thus be (partially) responsible for the toxicity of VirD5 and its induction of chromosome mis‐segregation as observed by Zhang *et al*. (2017). Aurora kinases share a highly homologous C‐terminal kinase domain, but have a variable N‐terminal regulatory domain. Using an *in vitro* pull‐down assay we found that VirD5 interacted with the highly conserved C‐terminal catalytic domain of yeast Aurora B/Ipl1 kinase, but not with the variable N‐terminal part (Fig. 1c). This suggested that VirD5 might also interact with the Aurora kinases from other organisms. In plants, the natural hosts for *Agrobacterium*‐mediated transformation, there are three Aurora kinases, called Aurora1, Aurora2 and Aurora3. To test whether VirD5 can interact with these plant kinases, we performed a BIFC experiment in *A. thaliana* protoplasts. A robust YFP fluorescence was seen in the nuclei of cells in which both VirD5 and one of the Aurora kinases from *A. thaliana* were present, indicating that all three plant Aurora kinases had strong interactions with VirD5 in the nucleus (Fig. 2). By contrast, in control BIFC experiments with either VirD5 or an Aurora kinase alone the fluorescent signal was not reconstituted.

### The N‐terminus of VirD5 interacts with Ipl1 and Dam1 and interferes with spindle elongation and chromosome segregation

**Figure 3:**
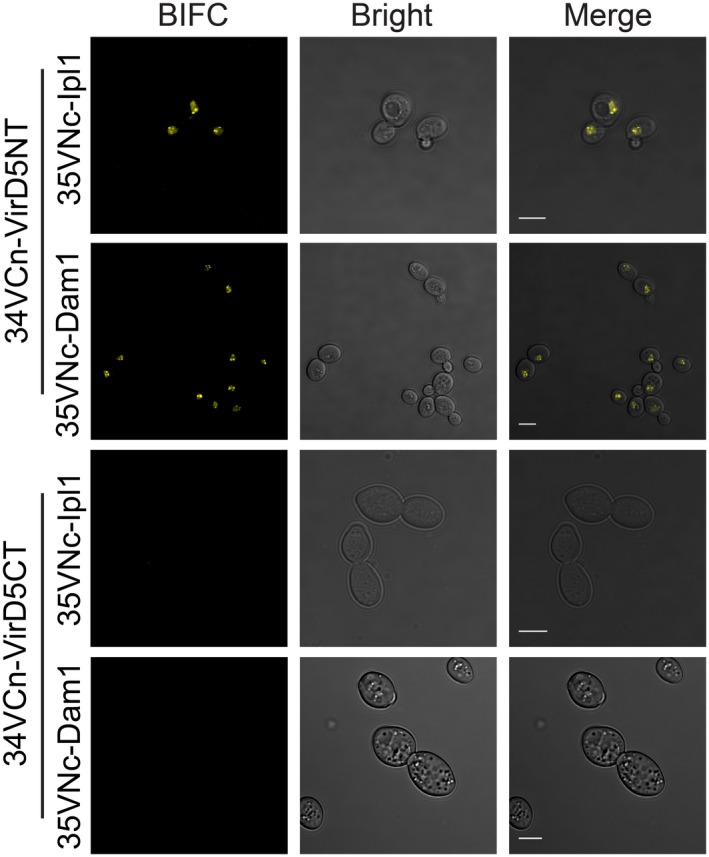
N‐terminus of VirD5 (VirD5NT) interacts with Dam1 and Ipl1. Yeast cells (BY4743) were transformed with BIFC vectors. 34VCn, the C‐terminus of Venus (VC173) fused with the N‐terminus of testing proteins. 35VNc, the N‐terminus of Venus (VN173) fused with the C‐terminus of testing proteins. VirD5NT, the N‐terminal 505 aa of VirD5. VirD5CT, the C‐terminal 313 aa of VirD5. Bars, 5 μm.

To determine which part of VirD5 binds to the Ipl1 kinase and the outer kinetochore/microtubule‐associated protein Dam1, we performed BIFC assays in yeast using several truncations of VirD5. In this way we found that the N‐terminal 505 aa of VirD5 (VirD5NT) gave strong interaction signals with both Ipl1 and Dam1, but not VirD5CT, the 313 C‐terminal amino acids (Fig. 3). Similar results were obtained when assayed in plant protoplasts (Fig. S6).

**Figure 4:**
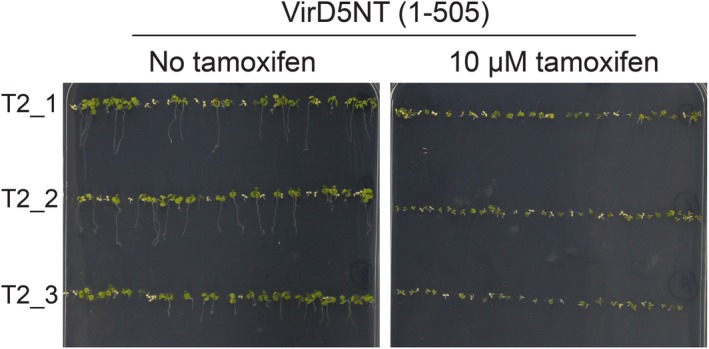
VirD5NT affects the growth of *Arabidopsis thaliana*. Three individual T2 heterozygous transgenic *A. thaliana* lines containing *virD5NT* behind the tamoxifen inducible promoter were grown on MS medium containing kanamycin with either DMSO or 10 μM tamoxifen dissolved in DMSO.

**Figure 5:**
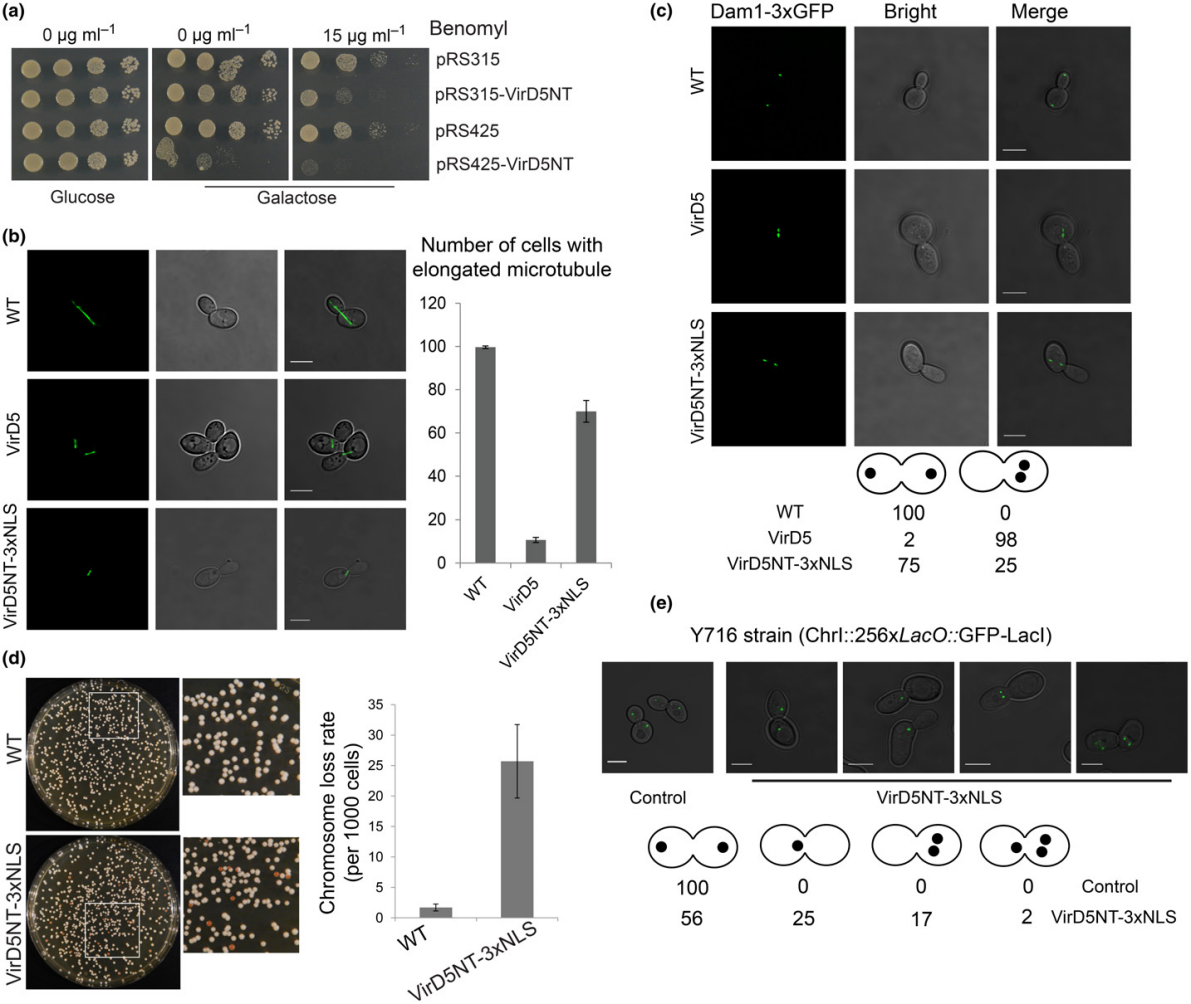
VirD5 affects microtubule elongation. (a) Yeast (BY4743) cells were transformed with either empty single‐copy plasmid (pRS315) or high‐copy plasmid (pRS425) or derivatives encoding VirD5NT driven by the *GAL1* promoter. Transformants were serially diluted five‐fold and spotted onto selection media containing either 2% glucose or 2% galactose with or without benomyl. (b) Tubulin‐GFP marked MAS101 cells and derivatives expressing VirD5 or VirD5NT‐3xNLS were grown first in YP rich medium containing 2% glucose and then shifted to YP medium containing 2% raffinose and 2% galactose for an additional 6 h. The spindle length of 100 mitotic cells was examined for each strain. Error bars represent the mean ± SD from three independent experiments. (c) Kinetochore/microtubule‐associated Dam1‐3xGFP marked yeast cells either expressing VirD5 or VirD5NT‐3xNLS were grown first in YP rich medium containing 2% glucose and then were shifted to YP medium containing 2% raffinose and 2% galactose for an additional 6 h. GFP dots were visualized under the confocal microscope. One hundred mitotic cells from each strain were observed. (d) Yeast strain RLY4029 forms white colonies, but upon loss of the minichromosome they turn red (Chen *et al*., 2012). Error bars represent the mean ± SD from three independent experiments. (e) The yeast strain Y716 contains a 256‐aa repeat *lacO* array in chromosome I and expresses GFP‐LacI. Wild‐type cells transformed with either empty plasmid (pRS425‐HYG) or its derivative (pRS425‐HYG‐VirD5NT‐3xNLS) encoding VirD5NT‐3xNLS under control of the *GAL1* promoter were first cultured in YP rich medium containing 2% glucose and hygromycin, and then shifted to YP rich medium containing hygromycin, 2% raffinose and 2% galactose for an additional 6 h. One hundred mitotic cells from each experiment were observed. Bars, 5 μm.

The VirD5NT part is toxic for yeast and plants (Fig. 4), although less toxic than the complete protein (Zhang *et al*., 2017). Therefore, we examined whether expression of VirD5NT in yeast cells increases their sensitivity to benomyl, a microtubule‐depolymerizing drug for which kinetochore mutants are hypersensitive. As can be seen in Fig. 5(a), control yeast cells transformed with either empty single‐copy (pRS315) or high‐copy (pRS425) vector showed a mild sensitivity to benomyl. However, yeast cells expressing both high and low levels of VirD5NT were heavily compromised in growth in the presence of benomyl, indicative of benomyl hypersensitivity‐like kinetochore mutants. To visualize the microtubules, we used a strain in which tubulin was labeled with GFP (Straight *et al*., 1997). This allowed us to visualize elongation of the spindle microtubules in anaphase in wild‐type mother cells and follow its entry from the mother cell into the bud (daughter cell). In contrast to control cells, in cells expressing VirD5, > 80% of anaphase cells retained short spindle microtubules that had not managed to enter into the daughter cell (Fig. 5b). Anaphase cells expressing VirD5NT also showed short spindles, but only in 30% of these cells (Fig. 5b). During spindle elongation in anaphase the chromosomes are attached to the spindle via their kinetochores. They are directed by the spindle to the opposite sides of the cell so that one copy can remain in the mother cell and the other copy can enter the bud (daughter cell). To visualize the position of the chromosomes during spindle elongation, we used a strain in which the Dam1 protein was labelled with a 3×GFP tag at its C‐terminus. The Dam1 protein forms rings encircling the microtubules that translate the force generated by depolymerization of the microtubules into movement of chromosomes attached via the kinetochores (Franck *et al*., 2007). Anaphase cells showed two bright Dam1 dots, with eventually one in the mother and the other in the daughter cell (Fig. 5c, upper panel). In cells containing VirD5 or VirD5NT, however, both dots often remained close together in the mother cell in line with the spindle elongation defect. The two dots were eventually either distributed over mother and daughter cell as in the absence of VirD5 or lagged in the middle of the mother cell. Lagging chromosomes were seen in 98% of the cells expressing VirD5 and in 25% of the cells expressing VirD5NT (Fig. 5c, middle and lower panels). These results demonstrate that VirD5 interferes with spindle microtubule elongation and entrance of the spindle into daughter cells.

Chromosome segregation errors were seen in cells expressing VirD5 (Zhang *et al*., 2017). Here we tested whether the VirD5NT fragment, like full‐length VirD5, would lead to chromosome loss and chromosome mis‐segregation in yeast cells. We used yeast strain RLY4029 (Chen *et al*., 2012), containing a minichromosome (CF) with the *URA3* gene and the *SUP11* gene suppressing red pigment accumulation in strains with a chromosomal *ade2‐101* mutation. RLY4029 cells carrying CF produce white colonies, but after loss of CF they form red colonies (Fig. S2). RLY4029 cells with and without a construct encoding VirD5NT under control of the *GAL1* promoter were grown in minimal medium containing 2% glucose but lacking uracil first, followed by a shift to medium (with uracil) containing 2% raffinose and 2% galactose for 24 h. The induced cells were serially diluted and plated on medium containing 2% glucose in order to repress expression of VirD5NT. As can be seen in Fig. 5(d), a more than 10‐fold higher rate of minichromosome loss was observed in cells expressing VirD5NT compared with that in control cells. These data indicate that VirD5NT, like the full protein, causes chromosome instability. To study whether the presence of VirD5NT would also generate chromosome mis‐segregation, we used strain Y716 in which chromosome I was visualized by binding of GFP‐LacI to an array of *lacO* repeats (Meyer *et al*., 2013). When we examined the distribution of chromosome I in dividing yeast cells expressing VirD5NT, we found that *c*. 40% of cells expressing VirD5NT displayed chromosome mis‐segregation (Fig. 5e). In comparison, 97% mis‐segregation was seen previously in the strain expressing full‐length VirD5 (Zhang *et al*., 2017).

### VirD5 stimulates the kinase activity of Ipl1/Aurora on Dam1

**Figure 6:**
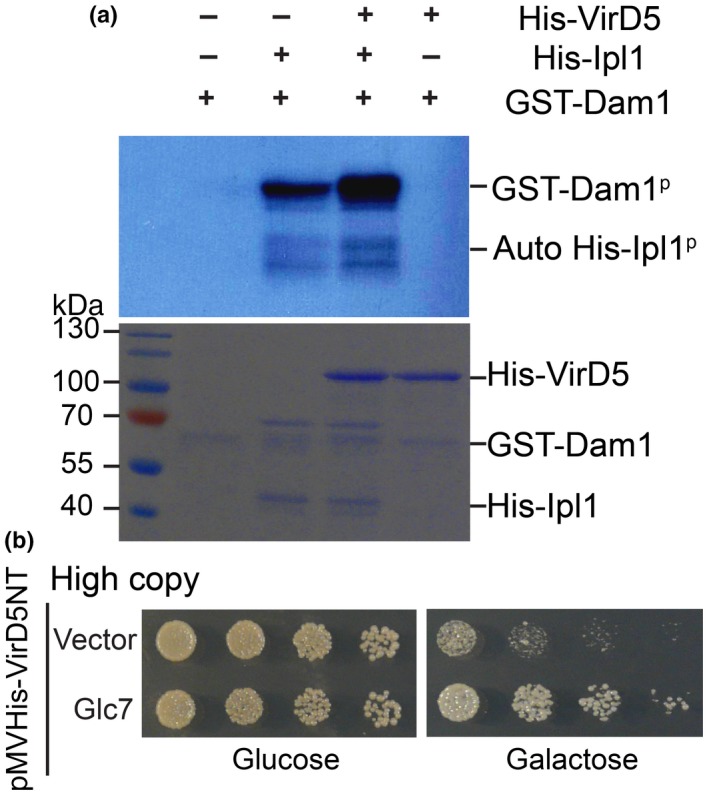
VirD5 stimulates the kinase activity of Ipl1. (a) GST‐tagged Dam1 purified from *Escherichia coli* was incubated with His‐tagged Ipl1 with or without His‐tagged VirD5 protein in kinase buffer containing [γ‐^32^P] ATP. The purified proteins used for the kinase assay were stained with Coomassie Brilliant Blue (CBB). (b) A high‐copy plasmid containing VirD5NT driven by the *GAL1* promoter (pMVHis‐VirD5NT) was cotransformed into haploid BY4741 cells either with a high‐copy empty plasmid (pRS425) or the same plasmid expressing phosphatase Glc7 from its own promoter and terminator. Transformants were serially diluted five‐fold and spotted onto selection medium containing either 2% glucose or 2% galactose.

Ipl1/Aurora kinase plays crucial roles in sensing and correcting erroneous kinetochore–spindle microtubule attachments by phosphorylating key substrates involved in the kinetochore–spindle binding. Both loss and overexpression of the Ipl1/Aurora kinases leads to massive chromosome mis‐segregation and aneuploidy in yeast cells (Chan & Botstein, 1993; Muñoz‐Barrera & Monje‐Casas, 2014 *)*. To determine whether VirD5 may affect the kinase activity of Ipl1, we carried out an *in vitro* kinase assay using the microtubule binding protein Dam1 as the substrate (Kang *et al*., 2001). GST‐Dam1 expressed and purified from *E. coli* was mixed with His‐tagged Ipl1 in the presence or absence of His‐tagged VirD5 in a phosphorylation buffer containing [γ‐^32^P] ATP; subsequently, the protein mixtures were separated on a SDS‐PAGE gel and analyzed by autoradiography. We observed a stronger ^32^P signal on the Dam1 substrate that had been incubated with Ipl1/Aurora kinase in the presence of VirD5 (Fig. 6a) than in control experiments in the absence of VirD5. The VirD5 protein itself showed no kinase activity on Dam1. This suggests that the toxicity of VirD5 may (partially) be explained by its stimulation of the activity of Ipl1/Aurora kinases.

### The toxicity of VirD5NT is suppressed by the Glc7 phosphatase *in vivo*

It has been found that protein phosphatase I (Glc7 in budding yeast) can oppose the kinase activity of Ipl1/Aurora kinase by dephosphorylating the same substrates to tightly regulate the cell cycle during mitosis (Francisco *et al*., 1994; Hsu *et al*., 2000; Pinsky *et al*., 2006). If the toxicity of VirD5 is indeed based on stimulation of the kinase activity of Ipl1, overexpression of the opposing phosphatase should be able to rescue cells from toxicity. To test this, we carried out a suppressor assay. As shown in Fig. 6(b), VirD5NT inhibited the growth of yeast cells, but this growth inhibition was indeed suppressed by overexpression of Glc7. This indicates that the increased kinase activity of Ipl1 does underly the toxicity of VirD5NT.

## Discussion

The *Agrobacterium* virulence protein VirD5 is one of the effector proteins which is translocated by the T4SS of the bacterium into host cells (Vergunst *et al*., 2005). It contributes to transformation in a way that is not yet completely understood, but in its absence transformation is reduced (Wang *et al*., 2018; Fig. S1). Expression of VirD5 in both plant and yeast cells inhibits growth and may cause cell death (Zhang *et al*., 2017). Yeast has been used successfully in studies of the virulence factors of animal and plant pathogens (Popa *et al*., 2016). We have used yeast as a model for studies of the molecular mechanism underlying the toxicity of VirD5. This showed that VirD5 is a nuclear protein binding to the kinetochores in the yeast nucleus. VirD5 is brought to the kinetochores via an interaction of its N‐terminal domain with the yeast Spt4 protein. In plant cells VirD5 is also a nuclear protein, which can interact with one of the plant Spt4 orthologs (Zhang *et al*., 2017). Here we found that VirD5 interacts in the yeast nucleus with both the Dam1 complex and Ipl1/Aurora kinase, when brought to the kinetochores through interaction with Spt4. The yeast Dam1 complex encircles the plus ends of microtubules (Miranda *et al*., 2005; Zelter *et al*., 2015), and is essential for bridging of the microtubules to the kinetochores through a direct interaction with the outer kinetochore protein Ndc80 (Tien *et al*., 2010; Lampert *et al*., 2013). The spindle separates the chromatids from each other by pulling them to the opposite spindle poles, but this occurs only after proper amphitelic attachment of the kinetochores of all the pairs of chromatids. Erroneous attachment of any of the kinetochores activates Ipl1/Aurora kinase, which phosphorylates outer kinetochore proteins of the Ndc80 complex and proteins of the Dam1 complex associated with the microtubules, which leads to detachment (Cheeseman *et al*., 2002; DeLuca *et al*., 2011; Sarangapani Krishna *et al*., 2015). When any of the kinetochores is not attached to a microtubule, the SAC is activated and only inactivated after proper attachment of all the kinetochores is realized (Sacristan & Kops, 2015). All this prevents chromosome mis‐segregation.

Using an *in vitro* assay we now found that VirD5 stimulates the activity of Ipl1/Aurora kinase leading to enhanced phosphorylation of the substrate Dam1. Moreover, the toxicity of VirD5NT could be suppressed *in vivo* by overexpression of the PP1 phosphatase Glc7, which acts antagonistically to Ipl1/Aurora kinase by dephosphorylating Ipl1 targets (Francisco *et al*., 1994; Hsu *et al*., 2000; Pinsky *et al*., 2006; Robinson *et al*., 2012). Increased Aurora kinase activity has been shown to cause continuous disruption of kinetochore–microtubule attachments, leading to constitutive activation of the SAC, spindle instability and defective chromosome segregation (Katayama *et al*., 2003; Vader & Lens, 2008; Demidov *et al*., 2014; Muñoz‐Barrera & Monje‐Casas, 2014). This is fully in agreement with our findings that VirD5 causes benomyl hypersensitivity, defective spindle elongation and chromosome mis‐segregation.

In the absence of VirD5, the transformation efficiency of *Agrobacterium* is reduced (Wang *et al*., 2018; Fig. S1). Expression of VirD5 in plant cells, however, induces chromosomal mis‐segregation (Zhang *et al*., 2017), which may lead to cell death and thus reduced transformation. We hypothesize that the temporary presence of low amounts of VirD5 in transformed cells may induce the SAC, allowing more time for DNA repair associated with T‐DNA integration before cytokinesis. Besides, in the context of tumorigenesis in plants by wild‐type *Agrobacterium*, chromosome mis‐segregation may contribute to the evolution of fast‐growing tumor cells and thus enhance tumor formation.

## Author contributions

XZ and PJJH designed the research; XZ performed the research; XZ and PJJH analyzed the data and wrote the paper.

## Supporting information

Please note: Wiley Blackwell are not responsible for the content or functionality of any Supporting Information supplied by the authors. Any queries (other than missing material) should be directed to the *New Phytologist* Central Office.

**Fig. S1** VirD5 contributes to transformation.
**Fig. S2** Rationale of chromosome loss assay.
**Fig. S3** Interaction of VirD5 with Dam1 depends on Spt4.
**Fig. S4** VirD5 from different bacterial species interacts with Dam1 and Ipl1.
**Fig. S5** Sequence alignment of Aurora kinases from different species.
**Fig. S6** N‐terminus of VirD5 (VirD5NT) interacts with Ipl1 in plant protoplasts.
**Table S1** Strains used in this study.
**Table S2** Plasmids used in this study.
**Table S3** Primers used in this study.
**Table S4** Candidate centromere/kinetochore proteins tested for interaction with VirD5.
