# Supplementary material for: The Agrobacterium VirD5 protein hyperactivates the mitotic Aurora kinase in host cells
Source: New Phytol. 2019 Mar 2;222(3):1551–60. doi: 10.1111/nph.15700 (PMC6667905; doi:10.1111/nph.15700)
Supplement: Supplementary file 1 — Fig. S1 VirD5 contributes to transformation. Fig. S2 Rationale of chromosome loss assay. Fig. S3 Interaction of VirD5 with Dam1 depends on Spt4. Fig. S4 VirD5 from different bacterial species interacts with Dam1 and Ipl1. Fig. S5 Sequence alignment of Aurora kinases from different species. Fig. S6 N‐terminus of VirD5 (VirD5NT) interacts with Ipl1 in plant protoplasts. Table S1 Strains used in this study. Table S2 Plasmids used in this study. Table S3 Primers used in this study. Table S4 Candidate centromere/kinetochore proteins tested for interaction with VirD5. [file NPH-222-1551-s001.pdf]

***New Phytologist* Supporting Information**

Article title: The Agrobacterium VirD5 protein hyper-activates the mitotic Aurora kinase in host cells

Authors: Xiaorong Zhang, and Paul J. J. Hooykaas

Article acceptance date: 13 January 2019

The following Supporting Information is available for this article:

Fig. S1 VirD5 contributes to transformation.

Fig. S2 Rationale of chromosome loss assay

Fig. S3 Interaction of VirD5 with Dam1 depends on Spt4.

Fig. S4 VirD5 from different bacterial species interacts with Dam1 and Ipl1.

Fig. S5 Sequence alignment of Aurora kinases from different species.

Fig. S6 N-terminus of VirD5 (VirD5NT) interacts with Ipl1 in plant protoplasts.

Table S1 Strains used in this study.

Table S2 Plasmids used in this study.

Table S3 Primers used in this study.

Table S4 Candidate centromere/kinetochore proteins tested for interaction with VirD5.

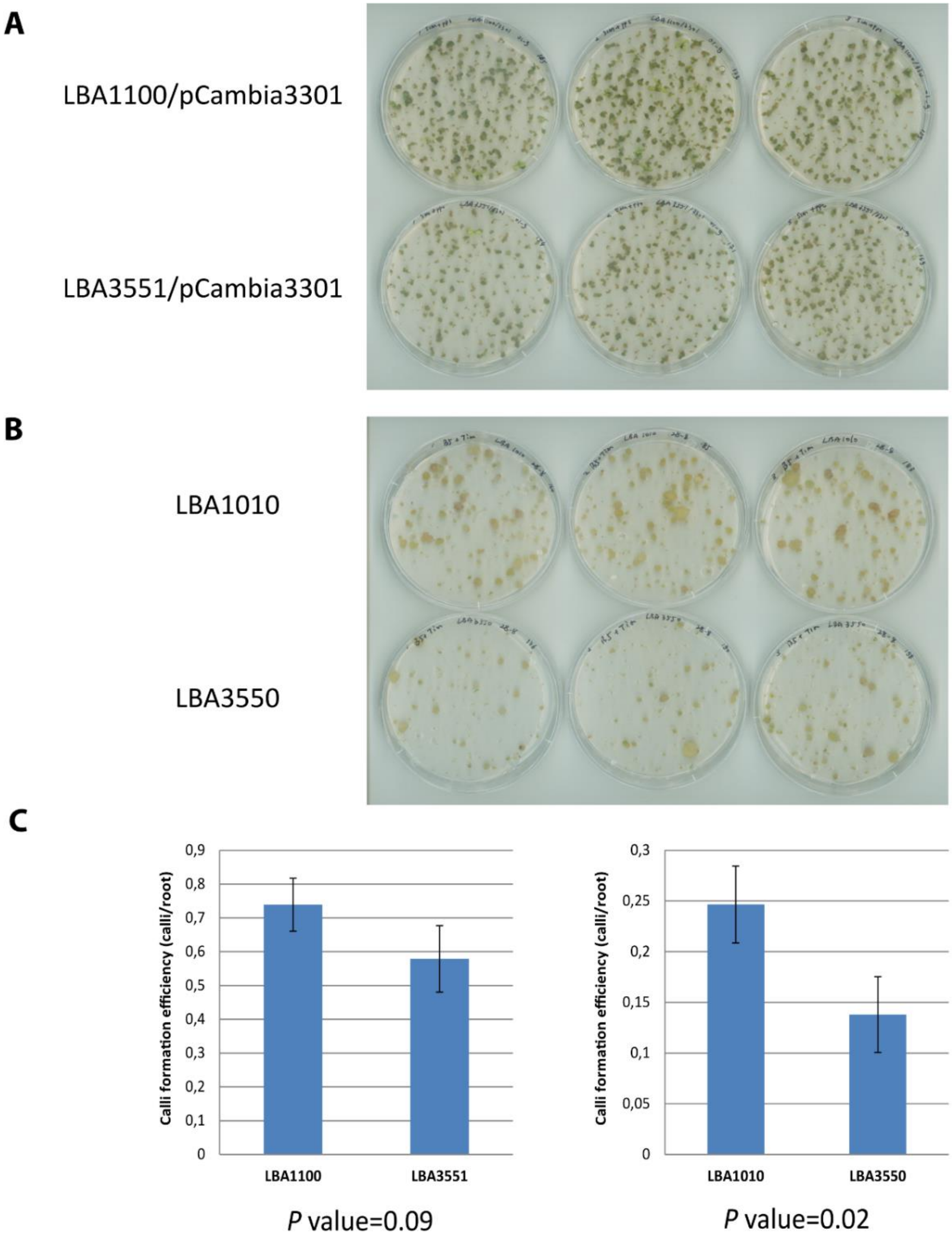

Fig. S1 VirD5 contributes to transformation. Transformation of *Arabidopsis* roots by helper strains LBA1100 and its *virD5* mutant (LBA3551), carrying binary vector pCAMBIA3301 (A), and by tumorigenic wild-type LBA1010 and the corresponding *virD5* mutant LBA3550 (B). A graphical representation of the results is given at the bottom (C). Error bars represent the mean +_SD from three independent experiments.

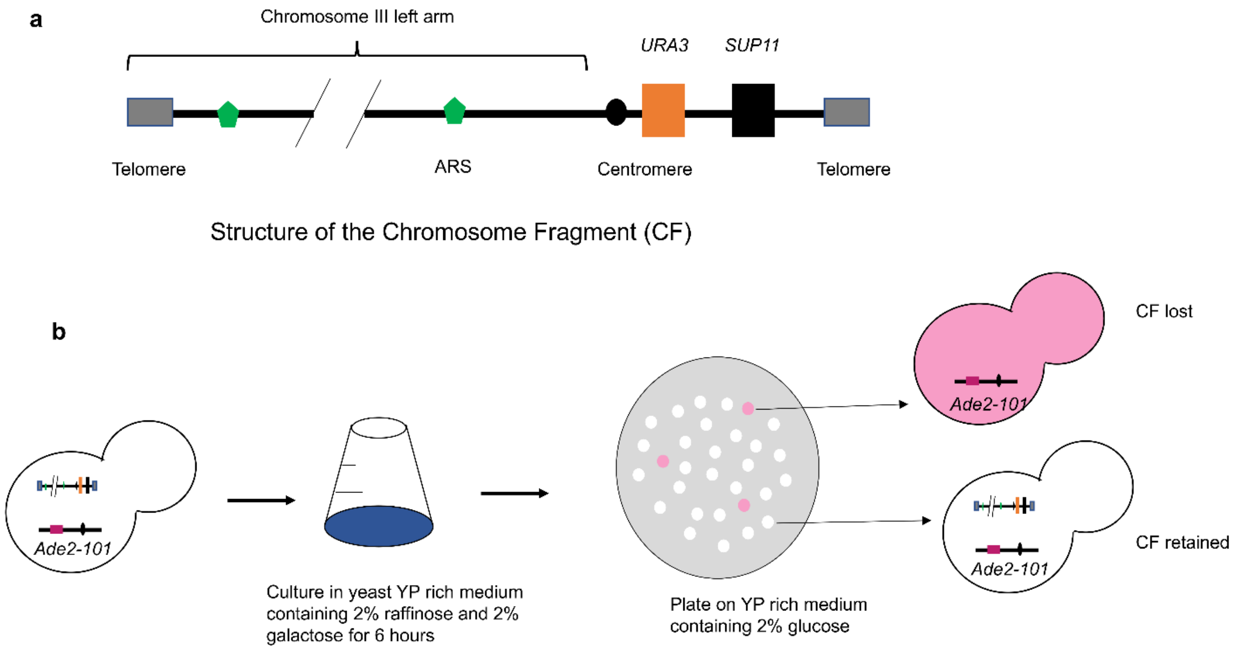

Fig. S2 Rationale of chromosome loss assay.

a. The structure of the mini-chromosome, which consists of a fragment of yeast Chromosome III, with the *SUP11* and *URA3* marker genes.

b. The haploid strain carries an *ade2-101* mutation and therefore forms red colonies in the absence of the mini-chromosome. The red pigment accumulation, however, is suppressed by the expression of *SUP11* present in the mini-chromosome, resulting in white colonies. The frequency of loss of this mini-chromosome can be calculated by counting the numbers of red colonies among the total numbers of colonies

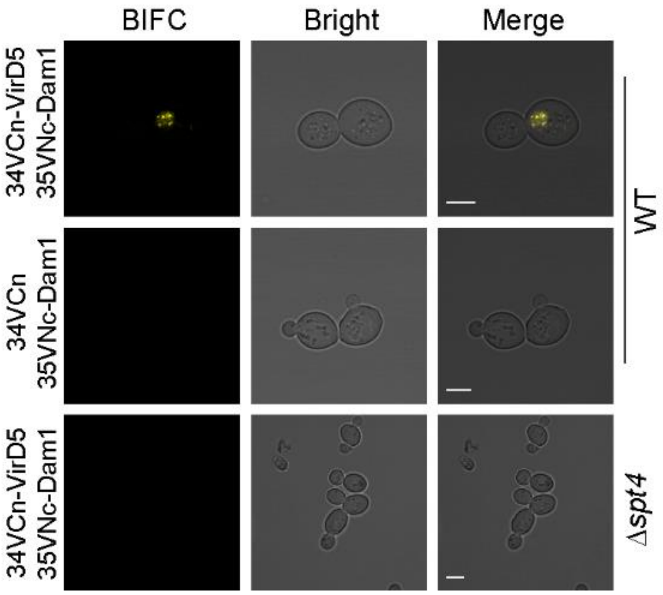

Fig. S3 Interaction of VirD5 with Dam1 depends on Spt4.

The wild type yeast strain BY4743 or the *spt4* mutant was transformed with BIFC vectors. 34VCn, the C-terminus of Venus (VC173) fused with the N-terminus of the proteins to be tested. 35VNc, the N-terminus of Venus (VN173) fused with the C-terminus of the proteins to be tested. Scale bars, 5 μm.

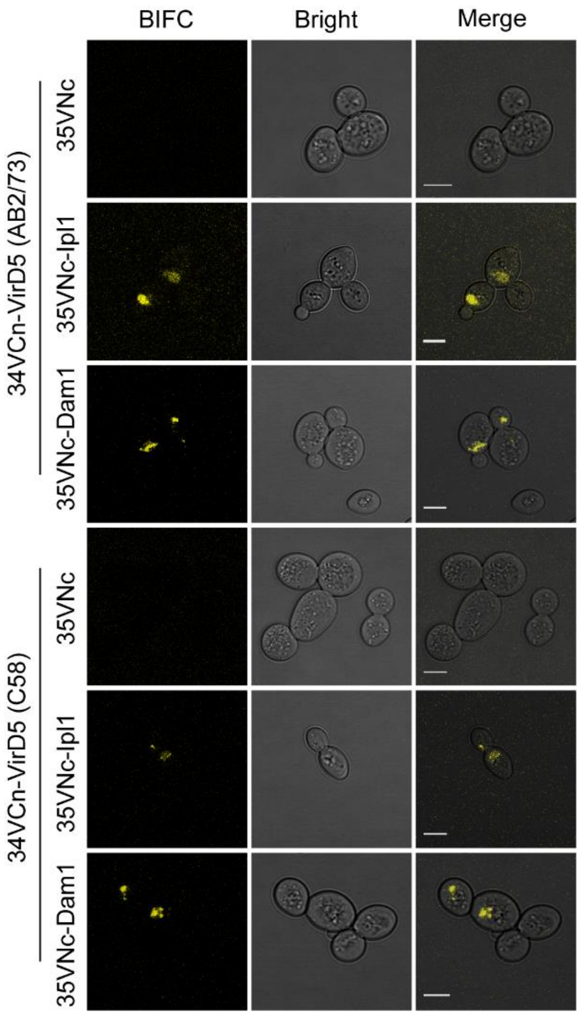

Fig. S4 VirD5 from different bacterial species interacts with Dam1 and Ipl1. VirD5 from the nopaline strain C58 and that from the limited-host range strain AB2/73 also interact with Ipl1 and Dam1. Yeast strain BY4743 was transformed with BIFC vectors. 34VCn, the C-terminus of Venus (VC173) fused with the N-terminus of the proteins to be tested. 35VNc, the N-terminus of Venus (VN173) fused with the C-terminus of the proteins to be tested. Scale bars, 5 µm.

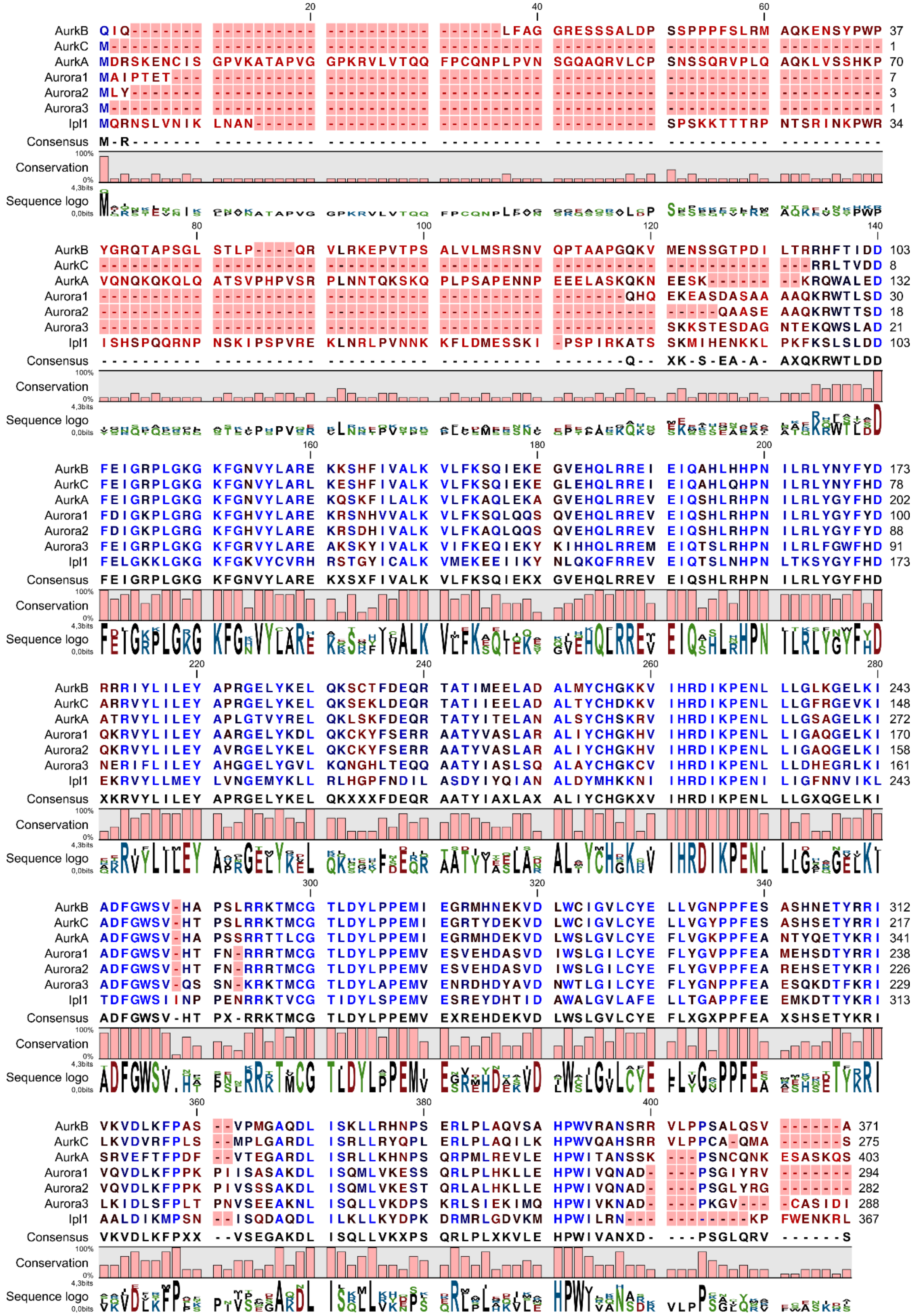
20 40 60
AurkB QIQ------- ---------- ---------- ------LFAG GRESSSALDP SSPPPFSLRM AQKENSYPWP 37
AurkC M--------- ---------- ---------- ---------- ---------- ---------- ---------- 1
AurkA MDRSKENCIS GPVKATAPVG GPKRVLVTQQ FPCQNPLPVN SGQAQRVLCP SNSSQRVPLQ AQKLVSSHKP 70
Aurora1 MAIPTET--- ---------- ---------- ---------- ---------- ---------- ---------- 7
Aurora2 MLY------- ---------- ---------- ---------- ---------- ---------- ---------- 3
Aurora3 M--------- ---------- ---------- ---------- ---------- ---------- ---------- 1
Ipl1 MQRNSLVNIK LNAN------ ---------- ---------- ---------- SPSKKTTTRP NTSRINKPWR 34
Consensus M-R------- ---------- ---------- ---------- ---------- ---------- ----------
Conservation
Sequence logo
80 100 120 140
AurkB YGRQTAPSGL STLP----QR VLRKEPVTPS ALVLMSRSNV QPTAAPGQKV MENSSGTPDI LTRRHFTIDD 103
AurkC ---------- ---------- ---------- ---------- ---------- ---------- ---RRLTVDD 8
AurkA VQNQKQKQLQ ATSVPHPVSR PLNNTQKSKQ PLPSAPENNP EEELASKQKN EESK------ --KRQWALED 132
Aurora1 ---------- ---------- ---------- ---------- -------QHQ EKEASDASAA AAQKRWTLSD 30
Aurora2 ---------- ---------- ---------- ---------- ---------- -----QAASE AAQKRWTTSD 18
Aurora3 ---------- ---------- ---------- ---------- ---------- SKKSTESDAG NTEKQWSLAD 21
Ipl1 ISHSPQQRNP NSKIPSPVRE KLNRLPVNNK KFLDMESSKI -PSPIRKATS SKMIHENKKL PKFKSLSLDD 103
Consensus ---------- ---------- ---------- ---------- -------Q-- XK-S-EA-A- AXQKRWTLDD
Conservation
Sequence logo
160 180 200
AurkB FEIGRPLGKG KFGNVYLARE KKSHFIVALK VLFKSQIEKE GVEHQLRREI EIQAHLHHPN ILRLYNYFYD 173
AurkC FEIGRPLGKG KFGNVYLARL KESHFIVALK VLFKSQIEKE GLEHQLRREI EIQAHLQHPN ILRLYNYFHD 78
AurkA FEIGRPLGKG KFGNVYLARE KQSKFILALK VLFKAQLEKA GVEHQLRREV EIQSHLRHPN ILRLYGYFHD 202
Aurora1 FDIGKPLGRG KFGHVYLARE KRSNHVVALK VLFKSQLQQS QVEHQLRREV EIQSHLRHPN ILRLYGYFYD 100
Aurora2 FDIGKPLGRG KFGHVYLARE KRSDHIVALK VLFKAQLQQS QVEHQLRREV EIQSHLRHPN ILRLYGYFYD 88
Aurora3 FEIGRPLGKG KFGRVYLARE AKSKYIVALK VIFKEQIEKY KIHHQLRREM EIQTSLRHPN ILRLFGWFHD 91
Ipl1 FELGKKLGKG KFGKVYCVRH RSTGYICALK VMEKEEIIKY NLQKQFRREV EIQTSLNHPN LTKSYGYFHD 173
Consensus FEIGRPLGKG KFGNVYLARE KXSXFIVALK VLFKSQIEKX GVEHQLRREV EIQSHLRHPN ILRLYGYFHD
Conservation
Sequence logo
220 240 260 280
AurkB RRRIYLILEY APRGELYKEL QKSCTFDEQR TATIMEELAD ALMYCHGKKV IHRDIKPENL LLGLKGELKI 243
AurkC ARRVYLILEY APRGELYKEL QKSEKLDEQR TATIIEELAD ALTYCHDKKV IHRDIKPENL LLGFRGEVKI 148
AurkA ATRVYLILEY APLGTVYREL QKLSKFDEQR TATYITELAN ALSYCHSKRV IHRDIKPENL LLGSAGELKI 272
Aurora1 QKRVYLILEY AARGELYKDL QKCKYFSERR AATYVASLAR ALIYCHGKHV IHRDIKPENL LIGAQGELKI 170
Aurora2 QKRVYLILEY AVRGELYKEL QKCKYFSERR AATYVASLAR ALIYCHGKHV IHRDIKPENL LIGAQGELKI 158
Aurora3 NERIFLILEY AHGGELYGVL KQNGHLTEQQ AATYIASLSQ ALAYCHGKCV IHRDIKPENL LLDHEGRLKI 161
Ipl1 EKRVYLLMEY LVNGEMYKLL RLHGPFNDIL ASDYIYQIAN ALDYMHKKNI IHRDIKPENI LIGFNNVIKL 243
Consensus XKRVYLILEY APRGELYKEL QKXXXFDEQR AATYIAXLAX ALIYCHGKXV IHRDIKPENL LLGXQGELKI
Conservation
Sequence logo
300 320 340
AurkB ADFGWSV-HA PSLRRKTMCG TLDYLPPEMI EGRMHNEKVD LWCIGVLCYE LLVGNPPFES ASHNETYRRI 312
AurkC ADFGWSV-HT PSLRRKTMCG TLDYLPPEMI EGRTYDEKVD LWCIGVLCYE LLVGYPPFES ASHSETYRRI 217
AurkA ADFGWSV-HA PSSRRTTLCG TLDYLPPEMI EGRMHDEKVD LWSLGVLCYE FLVGKPPFEA NTYQETYKRI 341
Aurora1 ADFGWSV-HT FN-RRRTMCG TLDYLPPEMV ESVEHDASVD IWSLGILCYE FLYGVPPFEA MEHSDTYRRI 238
Aurora2 ADFGWSV-HT FN-RRRTMCG TLDYLPPEMV ESVEHDASVD IWSLGILCYE FLYGVPPFEA REHSETYKRI 226
Aurora3 ADFGWSV-QS SN-KRKTMCG TLDYLAPEMV ENRDHDYAVD NWTLGILCYE FLYGNPPFEA ESQKDTFKRI 229
Ipl1 TDFGWSIINP PENRRKTVCG TIDYLSPEMV ESREYDHTID AWALGVLAFE LLTGAPPFEE EMKDTTYKRI 313
Consensus ADFGWSV-HT PX-RRKTMCG TLDYLPPEMV EXREHDEKVD LWSLGVLCYE FLXGXPPFEA XSHSETYKRI
Conservation
Sequence logo
360 380 400
AurkB VKVDLKFPAS --VPMGAQDL ISKLLRHNPS ERLPLAQVSA HPWVRANSRR VLPPSALQSV ------A 371
AurkC LKVDVRFPLS --MPLGARDL ISRLLRYQPL ERLPLAQILK HPWVQAHSRR VLPPCA-QMA ------S 275
AurkA SRVEFTFPDF --VTEGARDL ISRLLKHNPS QRPMLREVLE HPWITANSSK ---PSNCQNK ESASKQS 403
Aurora1 VQVDLKFPPK PIISASAKDL ISQMLVKESS QRLPLHKLLE HPWIVQNAD- ---PSGIYRV ------- 294
Aurora2 VQVDLKFPPK PIVSSSAKDL ISQMLVKEST QRLALHKLLE HPWIVQNAD- ---PSGLYRG ------- 282
Aurora3 LKIDLSFPLT PNVSEEAKNL ISQLLVKDPS KRLSIEKIMQ HPWIVKNAD- ---PKGV--- -CASIDI 288
Ipl1 AALDIKMPSN --ISQDAQDL ILKLLKYDPK DRMRLGDVKM HPWILRN--- --------KP FWENKRL 367
Consensus VKVDLKFPXX --VSEGAKDL ISQLLVKXPS QRLPLXKVLE HPWIVANXD- ---PSGLQRV ------S
Conservation
Sequence logo

Fig. S5 Sequence alignment of Aurora kinases from different species.

Protein sequences were from yeast Ipl1 (NP_015115), *Arabidopsis thaliana* Aurora 1 (NP_195009), Aurora 2 (NP_180159), Aurora 3 (NP_182073), and human AurkA (NP_003591), AurkB (NP_001271455) and AurkC (NP_001015878).

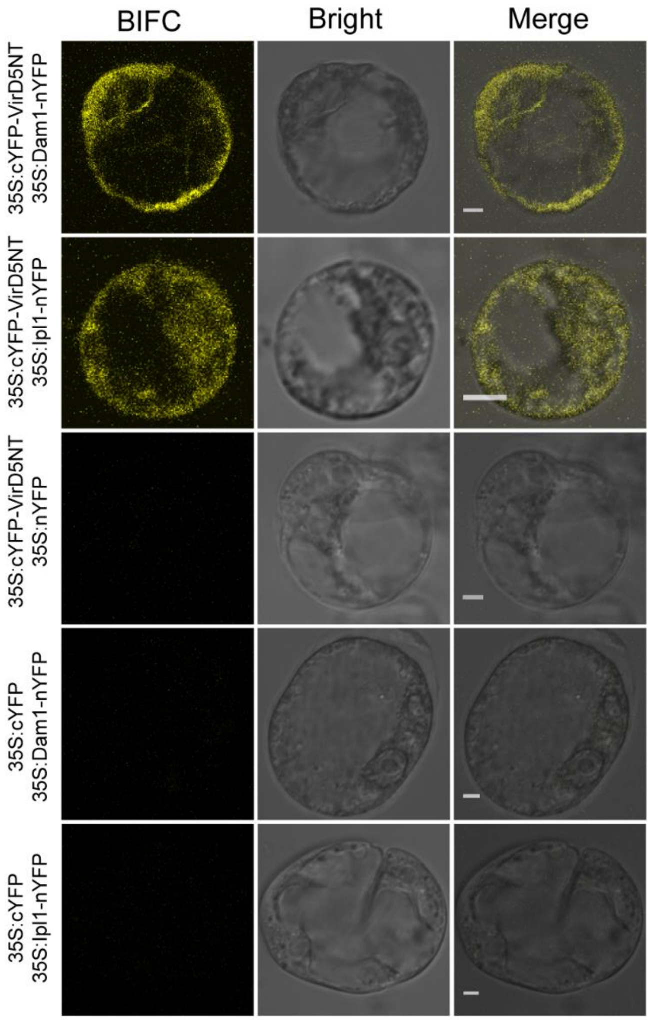

Fig. S6 N-terminus of VirD5 (VirD5NT) interacts with Ipl1 in plant protoplasts.

A. thaliana protoplasts were transformed with plant BIFC vectors. 35S, Cauliflower mosaic virus promoter. nYFP, N-terminus of YFP (1-154 aa). cYFP, C-terminus of YFP (155-238 aa). Scale bars, 5μm.

Table S1 Strains used in this study.

Name	Genotype and description	Source
BY4741	*MATa his3Δ1 leu2Δ0 met15Δ0 ura3Δ0*	(Brachmann *et al.*, 1998)
BY4741:Dam1-3xGFP	*MATa his3Δ1 leu2Δ0 met15Δ0 ura3Δ0; Dam1-3xGFP::KanMX* (PCR product mediated C-terminal endogeous fusion using pJET-3xGFP-KanMX as template)	This study
BY4741:Dam1-3xGFP:VirD5	*MATa his3Δ1 leu2Δ0 met15Δ0 ura3Δ0; Dam1-3xGFP::KanMX; pGAL1:VirD5::LEU2* (pRS305-pGAL1-VirD5 was integrated into the *LEU2* locus)	This study
BY4741:Dam1-3xGFP (pRS425-HYG)	*MATa his3Δ1 leu2Δ0 met15Δ0 ura3Δ0; Dam1-3xGFP::KanMX* (Episomal plasmid pRS425-HYG)	This study
BY4741:Dam1-3xGFP (pRS425-HYG-VirD5NT-3xNLS)	*MATa his3Δ1 leu2Δ0 met15Δ0 ura3Δ0; Dam1-3xGFP::KanMX* (Episomal plasmid pRS425-HYG-VirD5NT-3xNLS)	This study
BY4743	*MATa/α his3Δ1/his3Δ1 leu2Δ0/leu2Δ0 LYS2/lys2Δ0 met15Δ0/MET15 ura3Δ0/ura3Δ0*	(Brachmann *et al.*, 1998)
BY4743:VirD5	*MATa/α his3Δ1/his3Δ1 leu2Δ0/leu2Δ0 LYS2/lys2Δ0 met15Δ0/MET15 ura3Δ0/ura3Δ0; pGAL1:VirD5::LEU2* (pRS305-pGAL1-VirD5 was integrated into the *LEU2* locus)	This study
MAS101	*MATa ade2-1 can1-100 ura3-1 leu2-3,112 his3-11,15 trp1-1, GFP-TUB1-URA3*	(Straight *et al.*, 1997)
MAS101:VirD5	*MATa ade2-1 can1-100 ura3-1 leu2-3,112 his3-11,15 trp1-1, GFP-TUB1-URA3; pGAL1:VirD5::LEU2* (pRS305-pGAL1-VirD5 was integrated into the *LEU2* locus)	This study
MAS101 (pRS425-HYG)	*MATa ade2-1 can1-100 ura3-1 leu2-3,112 his3-11,15 trp1-1, GFP-TUB1-URA3* (Episomal plasmid pRS425-HYG)	This study
MAS101(pRS425-HYG-VirD5NT-3xNLS)	*MATa ade2-1 can1-100 ura3-1 leu2-3,112 his3-11,15 trp1-1, GFP-TUB1-URA3* (Episomal plasmid pRS425-HYG-VirD5NT-3xNLS)	This study
RLY-4029	(*MATa,ura3-52,lys2-801;ade2 101;trp1D1;leu2D1; +CFIII (CEN3.L.YFS2.1)URA3;SUP11;leu2D1*)	(Chen *et al.*, 2012)
RLY-4029 (pRS425-HYG)	(*MATa,ura3-52,lys2-801;ade2 101;trp1D1;leu2D1; +CFIII (CEN3.L.YFS2.1)URA3;SUP11;leu2D1*) (Episomal plasmid pRS425-HYG)	This study
RLY-4029 (pRS425-HYG-VirD5NT-3xNLS)	(*MATa,ura3-52,lys2-801;ade2 101;trp1D1;leu2D1; +CFIII (CEN3.L.YFS2.1)URA3;SUP11;leu2D1*) (Episomal plasmid pRS425-HYG-VirD5NT-3xNLS)	This study
Y716	*MATa leu2-? lys2::pLL1[PCYC1-GFP-lacI LYS2] met13-c tyr1-2, ura3-1 trp1-Δ63 cyh2-1 his3-Δ1 CEN1*::pJN2[*lacO256 LEU2*]	(Meyer *et al.*, 2013)

Y716 (pRS425-HYG)	*MATa leu2-? lys2::pLL1[PCYC1-GFP-lacI LYS2] met13-c tyr1-2, ura3-1 trp1-Δ63 cyh2-1 his3-Δ1 CEN1*::pJN2[*lacO256 LEU2*] (Episomal plasmid pRS425-HYG)	This study
Y716:VirD5 (pRS425-HYG-VirD5NT-3xNLS)	*MATa leu2-? lys2::pLL1[PCYC1-GFP-lacI LYS2] met13-c tyr1-2 ura3-1 trp1-Δ63 cyh2-1 his3-Δ1 CEN1*::pJN2[*lacO256 LEU2*]*;* (Episomal plasmid pRS425-HYG-VirD5NT-3xNLS)	This study

Table S2 Plasmids used in this study.

Name	Description	Source
pCAMBIA3301	Agrobacterium binary vector for plant transformation, with bialophos/phosphinothricin resistance and kanamycin resistance and GUS genes	Cambia
pGPINTAM	Binary vector with a tamoxifen inducible promoter.	(Lindhout, 2008)
pGPINTAM-VirD5NT	*VirD5NT* (1-505) was amplified using primers VirD5#33 and VirD5#61, PCR product digested with NotI was inserted into NotI of pGPINTAMNotI.	This study
pMVHis	High-copy yeast plasmid with a *GAL1* promoter and a *URA3* marker for expression of 6xHis-tagged proteins.	(van Hemert, *et al.*, 2003)
pMVHis-3xNLS	3xNLS fragment was annealed using primers 3NLSF and 3NLSR and was digested with XhoI and Xba I, the digested fragment was inserted into XhoI/XbaI of pMVHis.	(Zhang *et al*, 2017)
pMVHis-VirD5NT-3xNLS	*VirD5NT* was amplified using primers VirD5#1 and VirD5#81, PCR product digested with XmaI and XhoI was inserted into XmaI/XhoI of pMVHis-3xNLS.	(Zhang *et al*, 2017)
pET16H	pBR322 based plasmid with an N-terminal10xHis tag under the control of the T7 promoter.	Novagen
pET16H-VirD5	*VirD5* was amplified using primers VirD5#21 and VirD5#21-2, PCR product digested with ClaI and XmaI was inserted into ClaI/XmaI of pET16H.	(Zhang *et al*, 2017)
pET16H-Ipl1	*IPL1* was amplified using primers Ipl1HisFW and Ipl1HisREV, PCR product digested with ClaI and XmaI was inserted into ClaI/XmaI of pET16H.	This study
pGEX-KG	pMB1 based plasmid with an N-terminal GST tag under the control of the TAC promoter.	(Guan & Dixon, 1991)
pGEX-KG-Ipl1	*IPL1* was amplified using primers Ipl1FW and Ipl1REV, PCR product digested with SpeI and SalI was inserted into XbaI/SalI of pGEX-KG.	This Study
pGEX-KG-Ipl1NT	*IPL1NT* (1-118aa) was amplified using primers Ipl1NF and Ipl1NR, PCR product digested with XmaI and SalI was inserted into XmaI/SalI of pGEX-KG.	This Study
pGEX-KG-Ipl1CT	*IPL1CT* (101-367aa) was amplified using primers Ipl1CF and Ipl1CR, PCR product digested with XmaI and SalI was inserted into XmaI/SalI of pGEX-KG.	This Study
pGEX-KG-Dam1	*Dam1* was amplified using primers Dam1FW and Dam1REV, PCR product digested with NheI and SalI was inserted into XbaI/SalI of pGEX-KG.	This study
pUG34VCn	Single-copy plasmid with an N-terminal fusion with the C-terminal Venus part driven by the *MET25* promoter.	(Sakalis, 2013)
pUG34VCn-VirD5	*VirD5* was amplified using primers VirD5#38 and VirD5#23-2, PCR product digested with SpeI and SalI was inserted into SpeI/SalI of pUG34VCn.	(Zhang *et al*, 2017)

pUG34VCn-VirD5 (C58)	*VirD5* was amplified using primers C58-D5BIFCFW and C58-D5BIFCREV, PCR product digested with SpeI and SalI was inserted into SpeI of pUG34VCn.	This study
pUG34VCn-VirD5 (AB2/73)	*VirD5* was amplified using primers AB-D5BIFCFW and AB-D5BIFCREV, PCR product digested with SpeI and SalI was inserted into SpeI/SalI of pUG34VCn.	This study
pUG34VCn-VirD5NT	*VirD5NT* was amplified using primers VirD5#38 and VirD5#41, PCR product digested with SpeI and XhoI was inserted into SpeI/XhoI of pUG34VCn.	(Zhang *et al*, 2017)
pUG34VCn-VirD5CT	*VirD5CT* (521-833) was amplified using primers VirD5#74 and VirD5#23-2, PCR product digested with SpeI and SalI was inserted into SpeI/XhoI of pUG34VCn.	This study
pUG35VNc	Single-copy plasmid with a C-terminal fusion with the N-terminal Venus part driven by the *MET25* promoter.	(Sakalis, 2013)
pUG35VNc-Ipl1	*IPL1* was amplified using primers Ipl1FW and Ipl1REV, PCR product digested with SpeI and SalI was inserted into SpeI/SalI of pUG35VNc.	This study
pUG35VNc-Dam1	*Dam1* was amplified using primers Dam1FW and Dam1REV, PCR product digested with NheI and SalI was inserted into SpeI/SalI of pUG35VNc.	This study
pRS305	Yeast integrative plasmid with a *LEU2* marker.	(Sikorski & Hieter, 1989)
pRS305-pGAL1-VirD5	*pGAL1-His-VirD5-Ter* cassette was amplified using primers pMVHisVirD5FW and pMVHisVirD5REVand using pMVHis-VirD5 as template, PCR product digested with SpeI and SalI was inserted into SpeI/SalI of pRS305.	(Zhang *et al*, 2017)
pRS315	Single-copy yeast plasmid with a *LEU2* marker.	(Sikorski & Hieter, 1989)
pRS315-VirD5NT	*pGAL1-His-VirD5NT-Ter* cassette was amplified using primers pMVHisVirD5FW and pMVHisVirD5REVand using pMVHis-VirD5NT as template, PCR product digested with SpeI and SalI was inserted into SpeI/SalI of pRS315.	(Zhang *et al.*, 2017)
pRS425	Yeast high-copy plasmid with a *LEU2* marker.	(Christianson *et al.*, 1992)
pRS425-VirD5NT	*pGAL1-His-VirD5NT-Ter* cassette was amplified using primers pMVHisVirD5FW and pMVHisVirD5REVand using pMVHis-VirD5NT as template, PCR product digested with SpeI and SalI was inserted into SpeI/SalI of pRS425.	This study
pRS425-VirD5NT-3xNLS	*pGAL1-His-VirD5NT-3xNLS-Ter* cassette was amplified using primers pMVHisVirD5FW and pMVHisVirD5REVand using pMVHis-VirD5NT-3xNLS as template, PCR product digested with SpeI and SalI was inserted into SpeI/SalI of pRS425.	This study
pRS425-HYG	Hygromycin cassette containing promoter and terminator was cut off from pAG32 vector (Euroscarf) with *Not*I and was inserted into NotI of pRS425.	This study

pRS425-VirD5NT-3xNLS-HYG	Hygromycin cassette containing promoter and terminator was cut off from pAG32 vector (Euroscarf) with NotI and was inserted into NotI of pRS425-VirD5NT-3xNLS.	This study
pRS425-GLC7	*GLC7* including its own promoter and terminator was amplified using primers Glc7NFW and Glc7NREV, PCR product digested with XmaI and NotI was inserted into XmaI/NotI of pRS425.	This study
35S:cYFP (pSY735)	Plant BIFC vector with an N-terminal fusion with the C-terminus of YFP fragment (155-238 aa).	(Bracha-Drori *et al.*, 2004)
35S:cYFGP-VirD5	VirD5 was amplified using primers VirD5#15 and VirD5#24, PCR product digested with SalI and SpeI was ligated into SalI/SpeI of 35S:cYFP.	This study
35S:cYFGP-VirD5NT	VirD5NT was amplified using primers VirD5#15 and VirD5#87, PCR product digested with SalI and SpeI was ligated into SalI/SpeI of 35S:cYFP.	This study
35S:nYFP (pSY736)	Plant BIFC vector with an N-terminal fusion with the C-terminus of YFP fragment (1-154 aa).	(Bracha-Drori *et al.*, 2004)
35S:nYFP-Aurora1	*AURORA1* was amplified using primers Aurora1FW and Aurora1REV, PCR product digested with SalI and SpeI was inserted into SalI/SpeI of 35S:nYFP.	This study
35S:nYFP-Aurora2	*AURORA2* was amplified using primers Aurora2FW and Aurora2REV, PCR product digested with SalI and SpeI was inserted into SalI/SpeI of 35S:nYFP.	This study
35S:nYFP-Aurora3	*AURORA3* was amplified using primers Aurora3FW and Aurora3REV, PCR product digested with SalI and SpeI was inserted into SalI/SpeI of 35S:nYFP.	This study
35S:nYFP (pSY728)	Plant BIFC vector with an C-terminal fusion with the N-terminus of YFP fragment (1-154 aa).	Drori *et al.*, 2004)
35S:Ipl1-nYFP	Ipl1 was amplified using primers Ipl1-728F and Ipl1-728R, PCR product digested with NotI was ligated into NotI of 35S:nYFP (pSY728).	This study

Table S3 Primers used in this study.

Name	Sequence (5'-3')
AB-D5BIFCFW	GGACTAGTATGAAACCGTCAGGAAACT
AB-D5BIFCREV	ACGCGTCGAC TCATCGGCCGAAGCTCTCG
Aurora1FW	ACGCGTCGACAATGGCGATCCCTACGGAG
Aurora1REV	GGACTAGTTTAAACTCTGTAGATTCC
Aurora2FW	ACGCGTCGACAATGGGGATTTCTACAGAG
Aurora2REV	ACGCGTCGACAATGGGGATTTCTACAGAG
Aurora3FW	ACGCGTCGACAATGAGTAAGAAATCGACA
Aurora3REV	GGACTAGTTCAAATATCAATTGAGGC
C58-D5BIFCFW	GGACTAGTATGAGACCTTCAGGAAACCCG
C58-D5BIFCREV	GGACTAGTTCAGCGATTGAACGCTTTGT
Dam1FW	CATGCTAGCCATGAGCGAAGATAAAGCTAAAT
Dam1REV	ACGCGTCGACGTCTGAAGGGGGGCCTTGTA
Glc7NFW	CCGCCCGGGGGGAATCTGCAATTCTACAC
Glc7NREV	AAAGCGGCCGCTAGACTTATCATTTTTCCAC
Ipl1-728F	AAAGCGGCCGCATGCAACGCAATAGTTTAGTAAAT
Ipl1-728R	AAAGCGGCCGCGTTAACCGCTTATTTTCCCAAAAG
Ipl1HisFW	CCATCGATATGCAACGCAATAGTTTAG
Ipl1HisREV	CCCCCCGGGCTATAACCGCTTATTTTCCC
Ipl1FW	GGACTAGTCATGCAACGCAATAGTTTAGT
Ipl1REV	ACGCGTCGACGTAACCGCTTATTTTCCCAAAAGG
Ipl1NF	CCCCCCGGGAATGCAACGCAATAGTTTAG
Ipl1NR	ACGCGTCGACTTAAACTTTACCGAATTTACC
Ipl1CF	CCCCCCGGGACTCGATGACTTTGAACTGG
Ipl1CR	ACGCGTCGACCTATAACCGCTTATTTTCC
pMVHisVirD5FW	GGACTAGTTCACGCTGGGCGTAACCACCA
pMVHisVirD5REV	ACGCGTCGACATTAAAGCCTTCGAGCGTCCC
VirD5#1	CCGCCCGGGGATGACAGGAAAG
VirD5#1-2	CGCCTGCAGGACGGGATCGCTG
VirD5#15	GCGTCGACAATGACAGGAAAGTCG
VirD5#23-2	ACGCGTCGACTCAGCGTTTAAAC
VirD5#24	GGACTAGTTCAGCGTTTAAACGCT
VirD5#33	AAAGCGGCCGCAAACAGGAAAGTCGAAAGTTC
VirD5#38	GGACTAGTATGACAGGAAAGTCGA
VirD5#40	CCGCTCGAGTCAACCATATGCAGAAC
VirD5#41	CCGCTCGAGTCAGACGGGATCGCTG
VirD5#52	TGCTCTAGATTAGCGTTTAAACGCTTTGTC
VirD5#61	AAAGCGGCCGCTCAGACGGGATCGCTG
VirD5#74	GGACTAGTACCGTTACAGCTTCTATCC
VirD5#87	GGACTAGTTCAGACGGGATCGCTG

Table S4 Candidate centromere/kinetochore proteins tested for interaction with VirD5.

Protein name	Biological function	Interaction[a]
Ndc10	Inner kinetochore	N
Cep3	Inner kinetochore	N
Ctf13	Inner kinetochore	N
Cse4	Inner kinetochore	N
Mif2	Inner/middle kinetochore	N
Skp1	Inner kinetochore	N
Cbf1	Inner kinetochore	N
Ndc80	Outer kinetochore	N
Dam1	Outer kinetochore/Microtubule associated protein	**Y**
Ipl1	MT-Kinetochore regulatory aurora kinase	**Y**
Scc1	Cohesin	N
Esp1	Separase	N
Mad2	SAC checkpoint	N
Mps1	SAC checkpoint	N

a: N stands for no interaction; Y stands for interaction. Tested by BIFC.

References

Bracha-Drori K, Shichrur K, Katz A, Oliva M, Angelovici R, Yalovsky S, Ohad N. 2004. Detection of protein–protein interactions in plants using bimolecular fluorescence complementation. *Plant J* **40:** 419–427

Brachmann CB, Davies A, Cost GJ, Caputo E, Li J, Hieter P, Boeke J.D. 1998. Designer deletion strains derived from Saccharomyces cerevisiae S288C: A useful set of strains and plasmids for PCR-mediated gene disruption and other applications. *Yeast* **14:** 115–132.

Chen G, Bradford WD, Seidel CW, Li R. 2012. Hsp90 stress potentiates rapid cellular adaptation through induction of aneuploidy. *Nature* **482:** 246–250.

Christianson TW, Sikorski RS, Dante M, Shero JH, Hieter P. 1992. Multifunctional yeast high-copy-number shuttle vectors. *Gene* **110:** 119–122.

Guan K, Dixon JE. 1991. Eukaryotic proteins expressed in Escherichia coli: An improved thrombin cleavage and purification procedure of fusion proteins with glutathione S-transferase. *Analytical Biochemistry* **19:** 262–267.

Lindhout BI. 2008. *Regulatory DNA binding peptides as novel tools for plant functional genomics.* PhD thesis, Leiden University, Leiden, The Netherlands.

Meyer RE, Kim S, Obeso D, Straight PD, Winey M, Dawson DS. 2013. Mps1 and Ipl1/Aurora B act sequentially to correctly orient chromosomes on the meiotic spindle of budding yeast. *Science* **339:** 1071–1074.

Sakalis PA. 2013. *Visualizing virulence proteins and their translocation into the host during Agrobacterium -Mediated Transformation.* PhD thesis Leiden University, Leiden University, Leiden, The Netherlands.

Sikorski RS, Hieter P. 1989. A system of shuttle vectors and yeast host strains designed for efficient manipulation of DNA in Saccharomyces cerevisiae. *Genetics* **122:** 19–27.

Straight AF, Marshall WF, Sedat JW, Murray AW. 1997. Mitosis in living budding yeast anaphase A but no metaphase plate. *Science* **277:** 574–578.

van Hemert MJ, Deelder AM, Molenaar C, Steensma HY, van Heusden GPH. 2003. Self-association of the spindle pole body-related intermediate filament protein Fin1p and its phosphorylation-dependent interaction with 14-3-3 proteins in yeast. *Journal of Biological Chemistry* **278:** 15049–15055.

Zhang X, van Heusden GPH, Hooykaas PJJ. 2017. Virulence protein VirD5 of Agrobacterium tumefaciens binds to kinetochores in host cells via an interaction with Spt4. *Proceedings of the National Academy of Sciences USA* **114:** 10238-10243.
